# Nationwide comprehensive epidemiological study of rare diseases in Japan using a health insurance claims database

**DOI:** 10.1186/s13023-022-02290-0

**Published:** 2022-03-28

**Authors:** Kota Ninomiya, Masahiro Okura

**Affiliations:** 1 Social Cooperation Program of IT Healthcare, Graduate School of Pharmaceutical Sciences, The University of Tokyo, Tokyo, Japan; 2 National Institute of Public Health, Saitama, Japan; 3 Tohoku University Graduate School of Medicine, Miyagi, Japan

**Keywords:** Rare diseases, Claim data, Cross-disease analysis, National rare disease plan, Prevalence, Epidemiology, Natural history, Population-based study, Japan

## Abstract

**Background:**

There are more than 7000 rare diseases, most of which have no specific treatment. Disease profiles, such as prevalence and natural history, among the population of a specific country are essential in determining for which disease to research and develop drugs. In Japan, disease profiles of fewer than 2000 rare diseases, called Nanbyo, have been investigated. However, non-Nanbyo rare diseases remain largely uninvestigated. Accordingly, we revealed the prevalence and natural history of rare diseases among the Japanese population. This cross-disease study is the first to analyze rare-disease epidemiology in Japan with high accuracy, disease coverage, and granularity.

**Method:**

We applied for permission to use the National Database of Health Insurance Claims and Specific Health Checkups of Japan (NDB), which covered 99.9% of public health insurance claims from hospitals and 97.9% from clinics as of May 2015. Then, we obtained 10 years of data on the number of patients of approx. 4500 rare diseases, by sex and age. We translated disease names and established correspondences between rare diseases in NDB and those in Orphanet. Accordingly, we compared the prevalence and natural history between them.

**Results:**

About 3000 diseases in NDB are included in Orphanet and other medical databases. The data indicates that even if the Nanbyo systems do not cover a rare disease, its patients survive in many cases. Regarding natural history, genetic diseases tend to be diagnosed later in Japan than in the West. The data shown in this research are available in the Additional file 1 and the website of NanbyoData.

**Conclusions:**

Our research revealed the basic epidemiology and natural history of Japanese patients with some rare diseases using a health insurance claims database. The results imply that the coverage of the present Nanbyo systems is inadequate for rare diseases. Therefore, fundamental reform might be needed to reduce unfairness between rare diseases. Most diseases in Japan follow a tendency of natural history similar to those reported in Orphanet. However, some are detected later, partly because fewer clinical genetic tests are available in Japan than in the West. Finally, we hope that our data and analysis accelerate drug discovery for rare diseases in Japan.

**Supplementary Information:**

The online version contains supplementary material available at 10.1186/s13023-022-02290-0.

## Background

More than 7000 diseases constitute what are collectively known as rare diseases worldwide, regardless of the various definitions of “rare” in each country. Although the number of patients with each rare disease is much smaller than that for common diseases, the total amounts to about 400 million people worldwide [1]. Therefore, rare diseases are now recognized as a significant global public health issue [2].

The rarity of each disease affects its therapeutic environment both directly and indirectly. One of the difficulties patients face is that most rare diseases have no treatment. Specifically, 95% of them have no approved treatment options, including drugs [3]. As this is a crucial problem for patients, several reasons for the lack of treatment options have been identified. In the earliest phase, scientific knowledge and understanding of rare diseases are inadequate to develop new treatments because there is insufficient basic research on rare diseases [4]. Even if new drug candidate compounds are discovered, there are still some obstacles to drug development in pharmaceutical companies. Disease profiles, such as prevalence and natural history, are necessary for zeroing in on one rare target disease among others. The prevalence makes it possible to calculate profitability, while natural history or disease severity can prioritize rare diseases for early treatment. Although any rare disease can be severe on its patients, fatal ones need treatment as soon as possible. Moreover, the disease profiles among the population of a specific country are essential in deciding whether to conduct research and apply for drug approval. Therefore, if such detailed information is insufficient, developing treatments could become challenging.

Japan has been taking measures against rare disease problems for a long time, and there are two kinds of policies related to rare diseases in Japan. One is an Act that defines the priority settings for orphan drugs. If the number of patients with a targeted disease is less than 50,000, orphan drugs or devices are eligible for preferential reviews by the authority [5, 6]. The other policy is a medical expense subsidy policy related to rare and intractable diseases in Japan, called Nanbyo. More specifically, the policy covers two kinds of Nanbyo systems, the specific chronic pediatric disease system and the designated intractable disease system. The former is designed for children who have rare chronic and severe pediatric diseases, including cancers [7]. The latter applies to those with a severe condition because of rare and intractable diseases; these patients are estimated to amount to fewer than 120,000 people (0.1% of the Japanese population) [8]. In both systems, an advisory board consisting of specialists decides which diseases are eligible according to the policies’ purposes in reference to candidate disease profiles reported by the official research groups instituted.

There are 762 and 333 diseases officially announced as eligible in the specific chronic pediatric and designated intractable disease systems, respectively, as of September 2021. However, most diseases have various subtypes, and the range of covered diseases, including them, was obscure. Therefore, Nanbyo Disease Ontology (NANDO) was created to reveal the range and its hierarchical structure in a constructed format to show the world the correspondences between Nanbyo and diseases recognized as rare in Orphanet and other international databases and then to enable international data sharing [9].

According to NANDO, more than 1000 diseases are within the scope of each system, and most of them have equivalent terms in international databases, such as Monarch disease ontology and Orphanet Rare Disease ontology [10, 11]. Given some overlapping in the systems, patients with any of approximately 1800 diseases can receive medical expense subsidies. As for these covered diseases, although the number of Nanbyo diseases is still much smaller than that of rare diseases worldwide, the research groups investigated the profiles of the diseases in the Japanese population. However, on the other hand, there is little basic information about Japanese patients with rare diseases outside of the Nanbyo systems, which we call “non-Nanbyo rare diseases.” The lack of information makes it difficult to conduct research and development in Japan. Consequently, Japan has come to be left behind in drug development for rare diseases, and patients might encounter difficulties that might be reduced in other countries [12]. Approximately 43% of orphan drugs approved in the United States until May 2019 are not approved in Japan. Clinical trials for 75% of them have yet to be conducted in Japan [13].

This research study aims at revealing rare disease profiles in the Japanese population, particularly the prevalence of non-Nanbyo rare diseases, using the National Database of Health Insurance Claims and Specific Health Checkups of Japan (NDB). To the best of our knowledge, it is the first cross-disease study to disclose the epidemiology of rare diseases in Japan, with the highest accuracy, disease coverage, and granularity. We expect these profiles to promote drug development for rare diseases in Japan.

## Material and methods

### Data sources

In our observational study, we used the National Database of Health Insurance Claims and Specific Health Checkups of Japan (NDB) to obtain the number of patients with rare diseases in Japan. NDB was developed by Japan’s Ministry of Health, Labour and Welfare and disclosed to researchers and public workers to realize medical cost optimization [14]. It contains all electronic receipt data of almost all the patients who received medical care services under the universal health insurance system, from 99% of hospitals and clinics in Japan as of April 2015 [15]. As handwritten claims are not included, the amount of claims data added has increased every year according to their digitalization since April 2011 [16]. Approximately 1.9 billion claims have been added annually as of April 2018. Even if the receipt data are electronic, the NDB does not contain those exempt from medical expenses, mainly due to welfare aid. The NDB includes clinical information on standardized medical treatment, dental treatment, and dispensing work with anonymized patient profiles, such as age, sex, and diseases. As it contains detailed enormous information despite de-identification, researchers need to determine specific data requests and submit a minute research protocol to the ministry before viewing the data itself [17]. Accordingly, it is impossible to carry out exploratory research.

We requested summary data on the rare diseases chosen, the number of which was kept to a minimum, for our purposes. After review by the NDB expert council to guarantee proper use and security, we made contract with the ministry. Then, we were obligated to analyze the provided data only in a pre-specified secure room, following the guidelines on the use of the NDB. This study was approved by the institutional review board at the University of Tokyo as well.

### Requests for the NDB data

According to NDB usage rules, the ministry provides minimum data for individual research. In addition, clinical information in the NDB is classified according to the standard code master. Therefore, we were required to choose rare diseases from a disease-code master for NDB, the ICD10-based Standard Disease-Code Master [18, 19]. The code master includes 23,939 diseases as of June 2019 (NDB diseases) and has no translation.

We began to omit diseases that could be judged as common or not rare by category in the code list. The exclusion criteria are provided below:

- Certain infectious and parasitic diseases (A00-B99 in ICD-10)
- Injury, poisoning, and certain other consequences of external causes (S00-T88 in ICD-10)
- External causes of morbidity (V00-Y99 in ICD-10)
- Factors influencing health status and contact with health services (Z00-Z99 in ICD-10).

Secondly, we included diseases that seem rare with high probability based on the general tendency of rare diseases. The inclusion criteria are as follows:

- Congenital malformations, deformations, and chromosomal abnormalities (Q00-99 in ICD-10)
- Diseases the names of which imply a genetic abnormality, congenital anomaly, or rarity.

Then, we included diseases covered by NANDO, Orphanet, and the Genetic and Rare Diseases Information Center (GARD). First, we translated rare diseases in Orphanet and GARD into Japanese using a machine translation system. Next, we automatically processed the character strings of the terms in Orphanet, GARD, and NANDO, which include Japanese Nanbyo systems and NDB diseases, to deal with inconsistent spelling and fluctuation. The primary process is shown below:

- Convert all alphanumeric characters and special characters into half-width
- Remove stop words and punctuations
- Standardize disease-related words, such as 遺伝, 遺伝的, and 遺伝学的, all of which practically mean “genetic”.

Then, we searched for NDB diseases with equivalent terms in NANDO, Orphanet, and GARD by automatic exact string matching and included them for request. In addition to the exactly matched diseases, we included related ones to reduce false negatives. We adopted the two methods shown below:

- Diseases subordinate to those with the exact match
- Diseases the names of which have some words in common with names of diseases with the exact match.

Finally, we manually added some diseases and confirmed the rarity of diseases that remained included after these procedures above. Consequently, we chose 4524 diseases as rare diseases from the standard disease-code master.

After these selection procedures, we made a second attempt at NDB disease mapping to those in Orphanet, GARD, and NANDO manually so that NDB could be internationally comparable and available. We translated 4524 diseases into English using the ICD-10 browser [20], an English–Japanese medical dictionary [21], and medical journals. Then, we manually looked for the exact correspondences between NDB diseases and those in the other databases.

As it is almost impossible to scrutinize all the 23,939 diseases and search for counterparts in Orphanet and GARD, we performed disease matching twice during the automatic narrowing-down process following the regulation and the manual matching process for availability.

### Number of patients

**Fig. 1:**
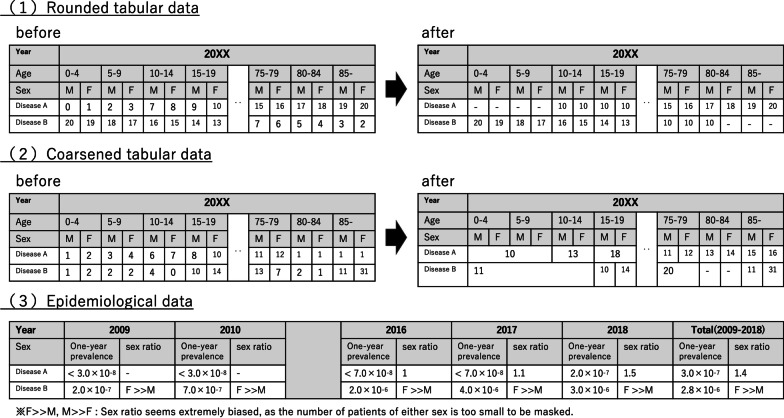
Schematic of disclosable tabular data and its masking

We requested the number of patients presenting with a total of 4524 diseases by sex and age for 2009 through 2018 and the total number by sex in those 10 years. The ministry takes care to avoid double counting in the data extraction procedure. Use of NDB data follows a data disclosure policy based on the cell size suppression policy of the U.S. Centers for Medicare & Medicaid Services, which stipulates that no cell containing a value of 1 to 10 can be reported directly [22]. Per NDB policy, we cannot disclose any cell showing the number of patients with a specific disease if it is from 0 to 9 at a specific year, sex, and age. Therefore, we made three kinds of tabular data based on the same provided data by masking them properly so as to obtain as much help from them as possible (Fig. 1).

The first table in Fig. 1 shows rounded tabular data, which contains the rounded-off number. As the regulation prohibits the value 0 from being disclosed, the actual value between 0 and 4 needs masking to “-.” This data shows rough trends in each disease even if extremely few people have it.

Then, the second table in Fig. 1 is coarsened tabular data made by a masking method of merging cells. For example, when male and female patients aged 10–14 with a particular disease at a specific year add up to 6 and 7, respectively, a total value of 13 can be disclosed by combining the two cells. As there are sometimes various merging patterns, we prioritized making open as many actual values as possible. It became possible with this data to know the status quo of rare diseases throughout Japan.

The third table is summary data with epidemiological parameters: prevalence and sex ratio. We calculated one-year and 10-year prevalence with the annual and total data, respectively. As in former studies [23–25], in calculating prevalence, we adopted the total Japanese population based on the Basic Resident Registration System published by the Ministry of Internal Affairs and Communications as denominator. We calculated the sex ratio with the actual number and the rounded number required. When a disease is too imbalanced to calculate its ratio, it is stated as “F ≫ M” or “M ≫ F.”

We also required the total number of patients to reveal natural history, such as the average age at onset and death. We counted the total patients by age and sex in 10 years, allowing double counting. For example, if patients survived for 10 years, they were mostly counted three times. We masked it in the two methods described above. The data reveal the diachronic trend of patients’ age and sex.

### Comparison of prevalence and natural history

We compared prevalence in NDB with that in Orphanet. First, we extracted the validated point prevalence in Orphanet from the Orphanet Epidemiological file as of October 2021 [27]. Then, we assigned six ordinal Orphanet prevalence categories (< 1/1,000,000; 1–9/1,000,000; 1–9/100,000; 1–5/10 000; 6–9/10 000; > 1/1000) to Japanese one-year prevalence values, which are calculated using the rounded values in 2018 and Japanese total population in 2018 [28].

We also compared each disease’s natural history, especially the age of onset and death. Orphanet divided the average onset timing into nine categories (Antenatal, Infancy, Neonatal, Childhood, Adolescent, Adult, Elderly, All ages, and No data available) and the average death timing into 13 categories (Embryofetal, Stillbirth, Infantile, Early childhood, Late childhood, Adolescent, Young adult, Adult, Elderly, Normal life expectancy, Any age, Not yet documented, and No data available). Each disease often has multiple categories.

As it takes much time to obtain a correct diagnosis in the field of rare diseases, the time of diagnosis in NDB does not mean the time of onset [29]. The average age at death is also difficult to identify because of a systemic flaw of NDB. Accordingly, we considered the summed number of all the living patients by age and by sex during the 10 years in NDB to be the diachronic age distribution pattern of patients. Then, we applied time-series clustering to the rounded number of patients between the ages of 0 and 84; we divided the diseases into five age distribution patterns (Congenital, Childhood, Acquired, Elderly, and Not applicable) based on silhouette analysis. We assigned the onset and death categories to the NDB age patterns from their trends, respectively.

## Results

### Data source

**Fig. 2:**
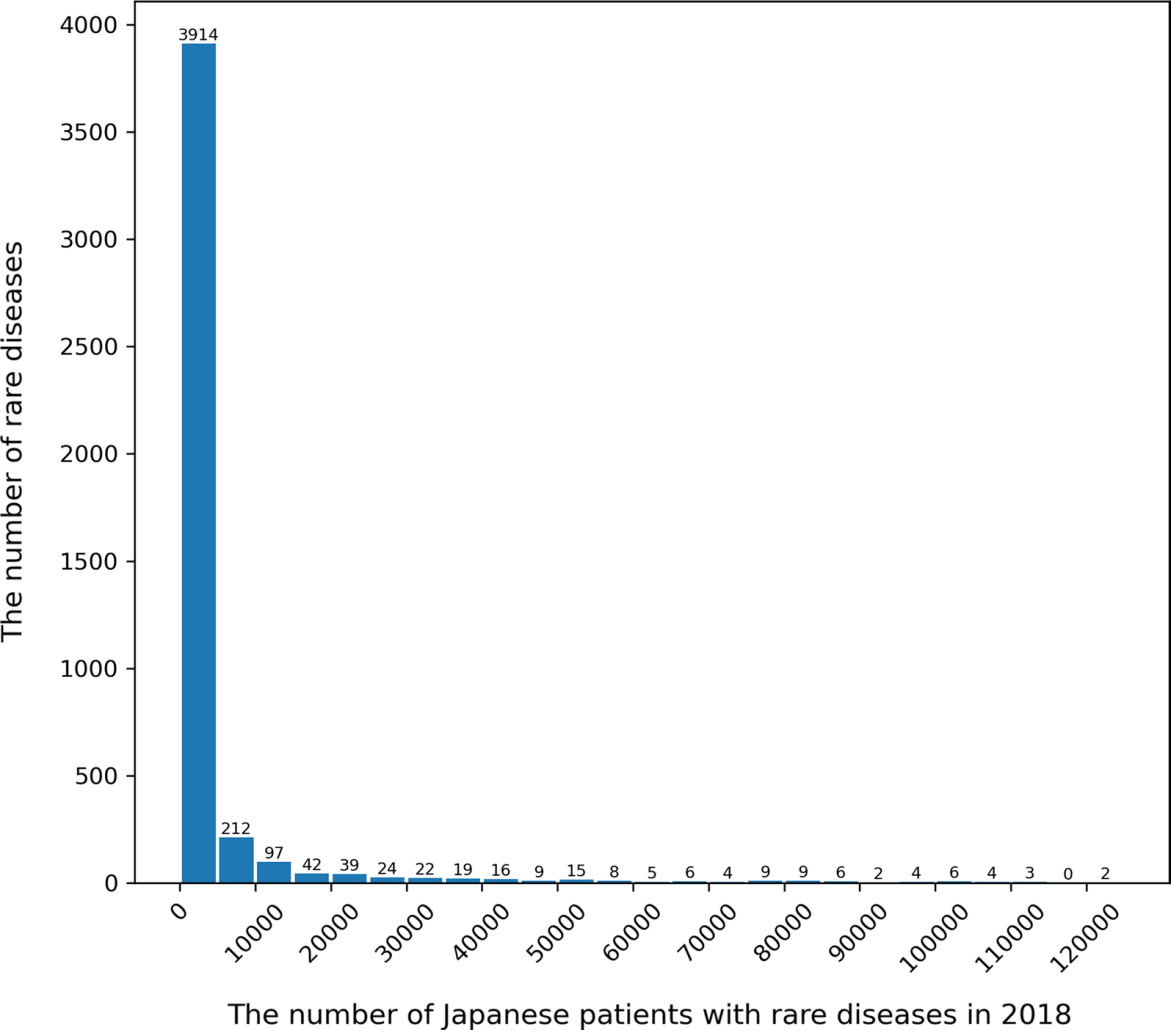
Distribution of the number of patients with selected diseases in NDB in 2018. The figure shows the number of rare diseases per the number of patients in 2018. As more recent data tends to be more accurate, the figure is generated based on the data in the most recent year, 2018

First, a histogram of the number of rare diseases per the number of patients in 2018 is ahown in Fig. 2. In addition, Fig. 2 depicts the comparison between the prevalence distribution in NDB and that in Orphanet, which is derived from the Orphanet report about bibliographical data [26], to validate the selection of rare diseases from the ICD10-based Standard Disease-Code Master.

**Fig. 3:**
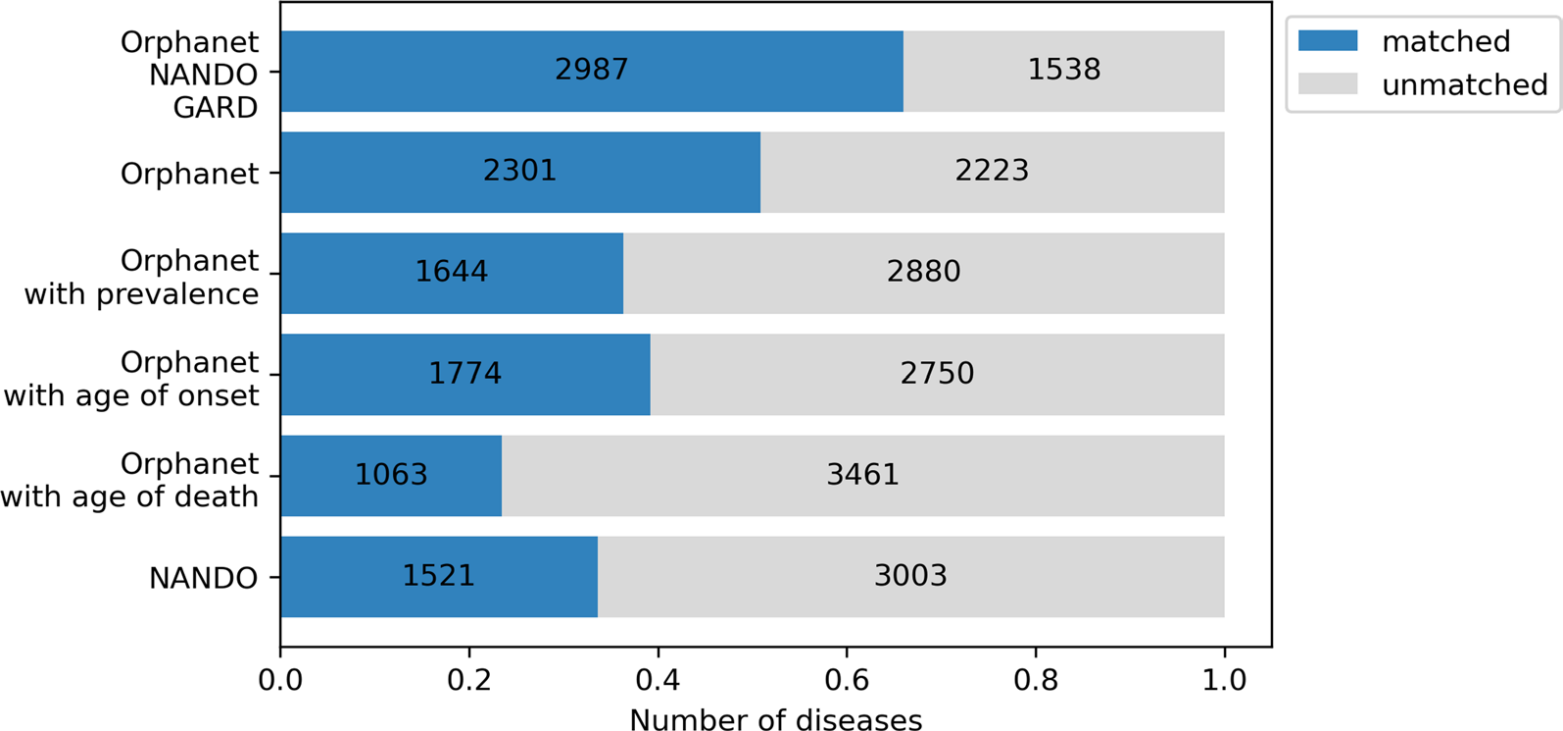
Number of diseases with/without correspondences. The top of the bar plot shows the number of the union of matched diseases in Orphanet, GARD, and NANDO. As some diseases in Orphanet have no epidemiological data or natural history, in addition to the second bar indicating disease-based direct comparison, the 3rd, 4th, and 5th figure illustrated the relationship between NDB diseases and Orphanet diseases whose prevalence, average age of onset, and average age of death is disclosed respectively. The bottom bar shows the correspondences between NDB diseases and NANDO diseases, which means Nanbyo

Figure 3 shows the number of diseases in NDB with/without the correspondence with several databases.

### Comparison of prevalence and natural history

**Fig. 4:**
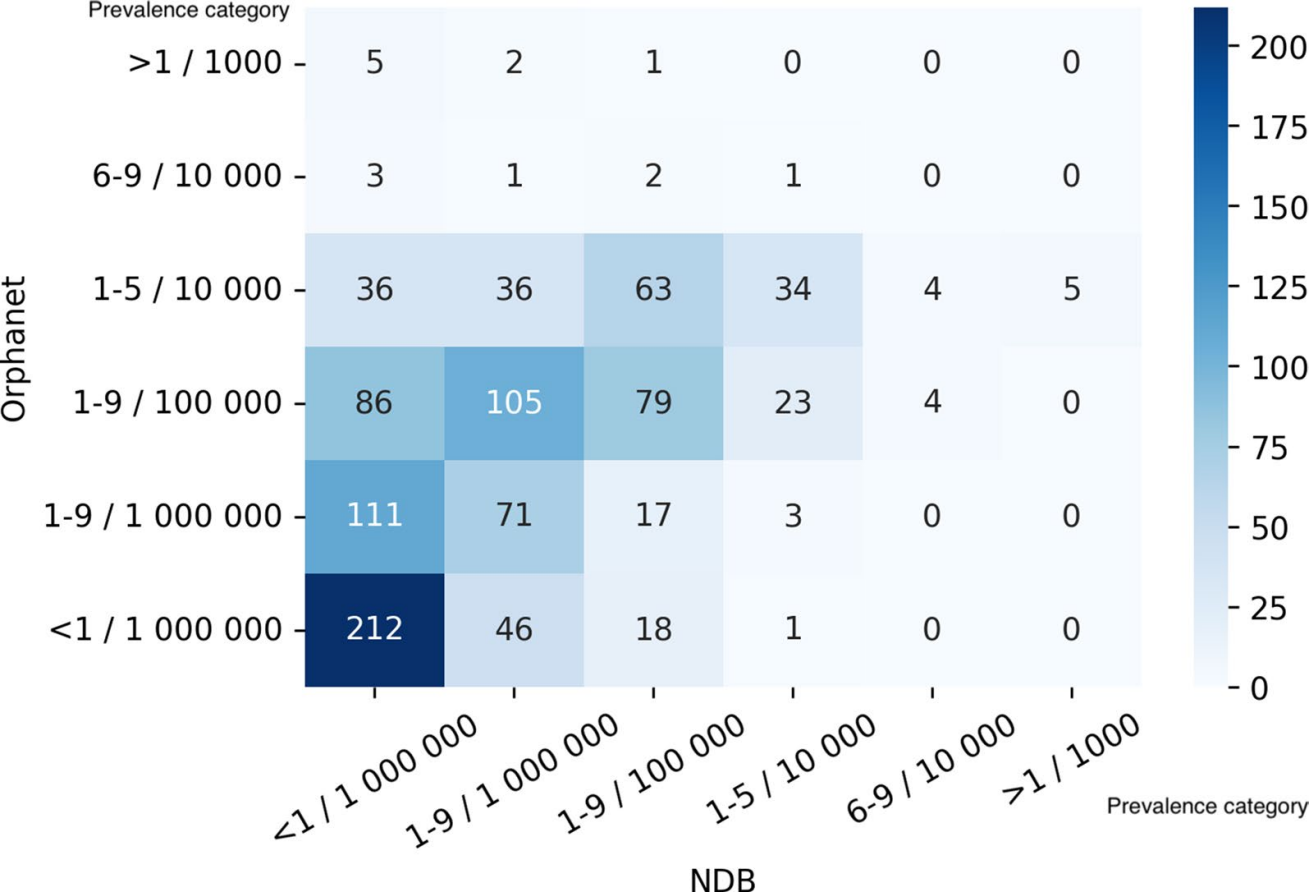
Heatmap of the number of diseases by prevalence category in Orphanet and NDB. The figure showed the relationship of the prevalence categories between Orphanet and NDB data. We omitted the values of “Specific population, “Not yet documented,” and “Unknown” in Orphanet. As Orphanet sometimes displays more than one prevalence category for a disease, we adopted a median category to represent comparability

**Fig. 5:**
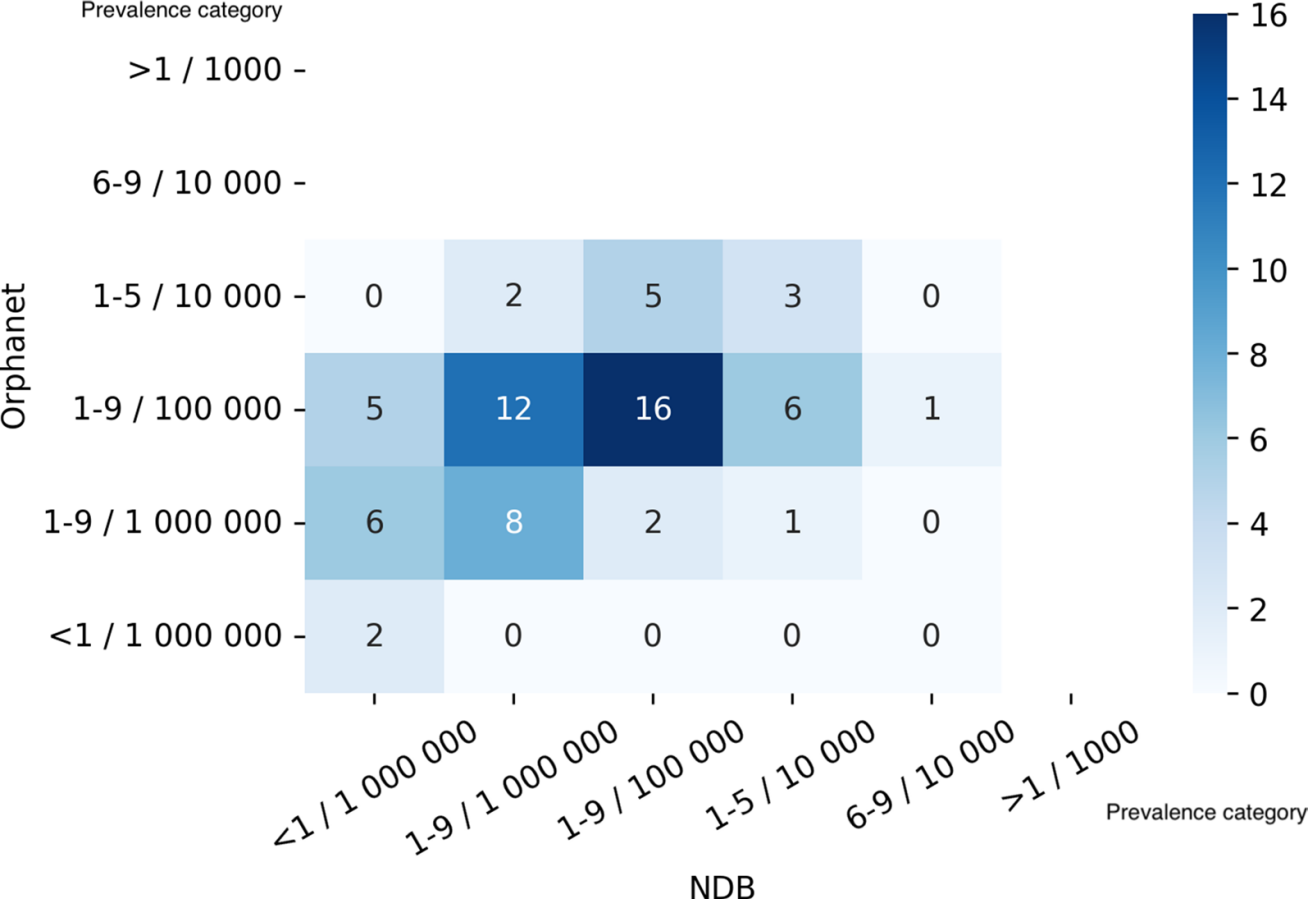
Heatmap of the number of diseases by prevalence category in Orphanet and NDB—Japanese patients. The figure showed the relationship focusing on Japanese data in Orphanet. We omitted the values of “Specific population, “Not yet documented,” and “Unknown” in Orphanet. As Orphanet sometimes displays more than one prevalence category for a disease, we adopted a median category to represent comparability

Figure 4 showed a heatmap of the number of diseases sorted by prevalence categories in NDB and Orphanet. We also noted a heatmap of the correspondence focusing on the validated Japanese point prevalence data in Orphanet in Fig. 5.

**Fig. 6:**
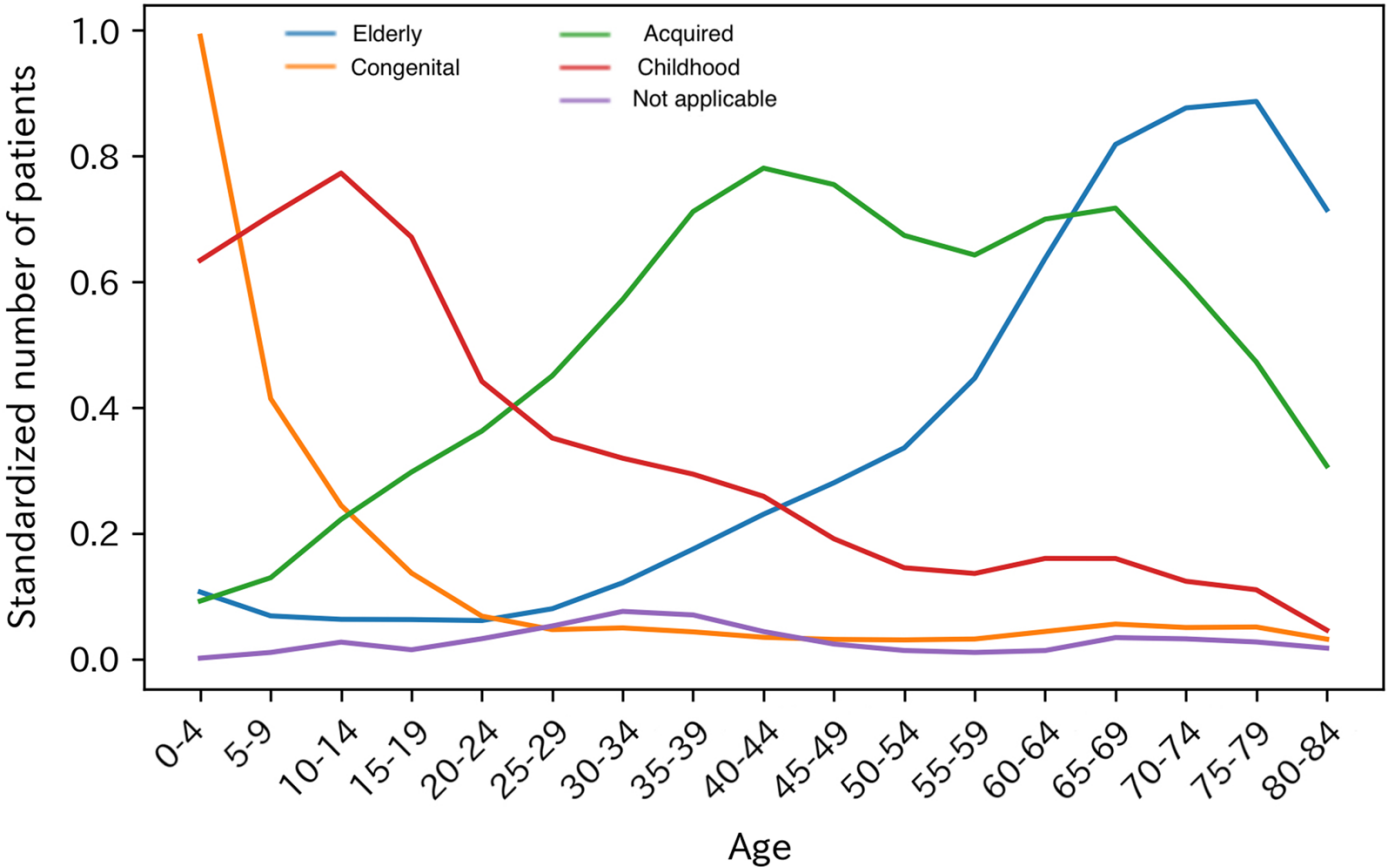
Age distribution patterns derived from time-series clustering. The figure illustrated the ideal patterns of each cluster (Congenital, Childhood, Acquired, Elderly, and Not applicable) based on the time-series analysis

**Fig. 7:**
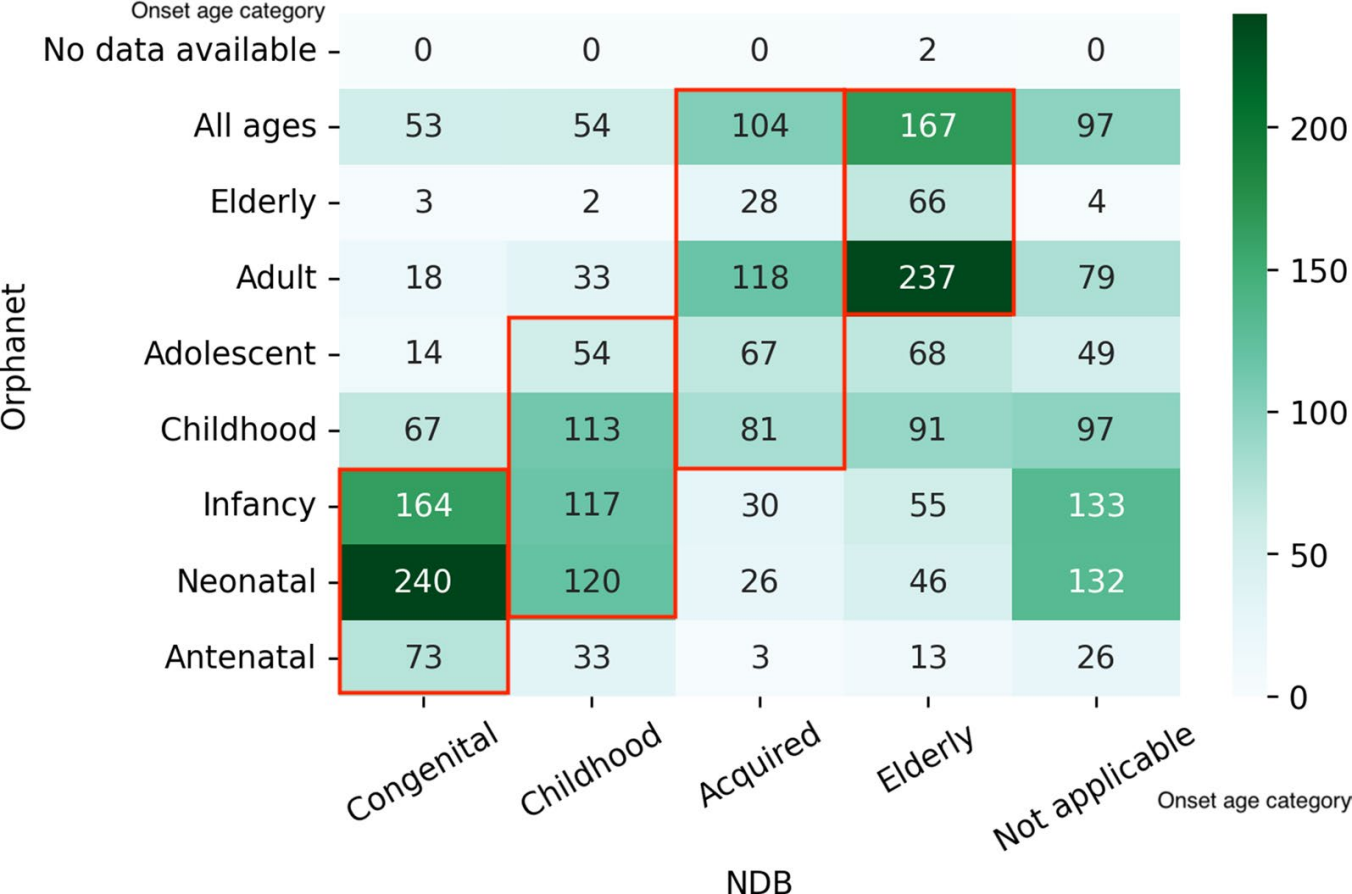
Heatmap of the number of diseases by age categories of onset in Orphanet and NDB. The figure shows the relationship between onset categories in Orphanet and the age distribution pattern based on NDB data. The red frames indicate predefined correspondence between both categories. The category “Not applicable” means there are too few patients suffering from a specific disease to calculate the age trend

**Fig. 8:**
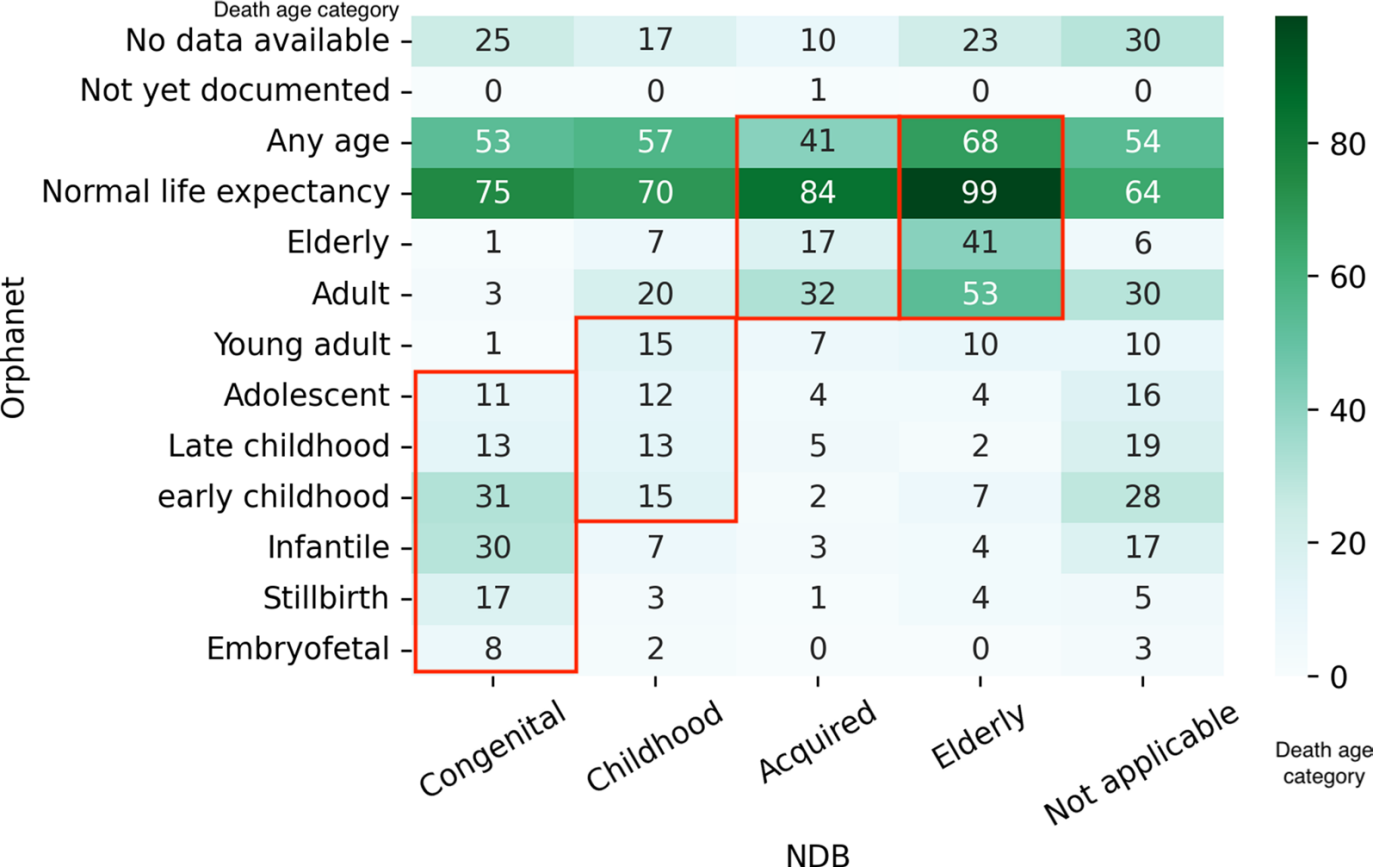
Heatmap of the number of diseases by age categories of death in Orphanet and NDB. The figure shows the relationship between death categories in Orphanet and age distribution patterns based on NDB data. The red frames indicate predefined correspondence between both categories. The category “Not applicable” means there are too few patients suffering from a specific disease to calculate the age trend

Then, we applied time-series clustering to the rounded number of patients between the ages of 0 and 84; we divided the diseases into five age distribution patterns (Congenital, Childhood, Acquired, Elderly, and Not applicable) based on the time-series analysis. Each age pattern is depicted in Fig. 6. We observed the comparison of the age categories in NDB with those in Orphanet for each disease, focusing on the average age of onset and death in Figs. 7 and 8 respectively.

We will disclose all these data in the Additional file 1 of this article. We will also make it searchable in NanbyoData (https://nanbyodata.jp) soon.

## Discussion

### Data validation

**Fig. 9:**
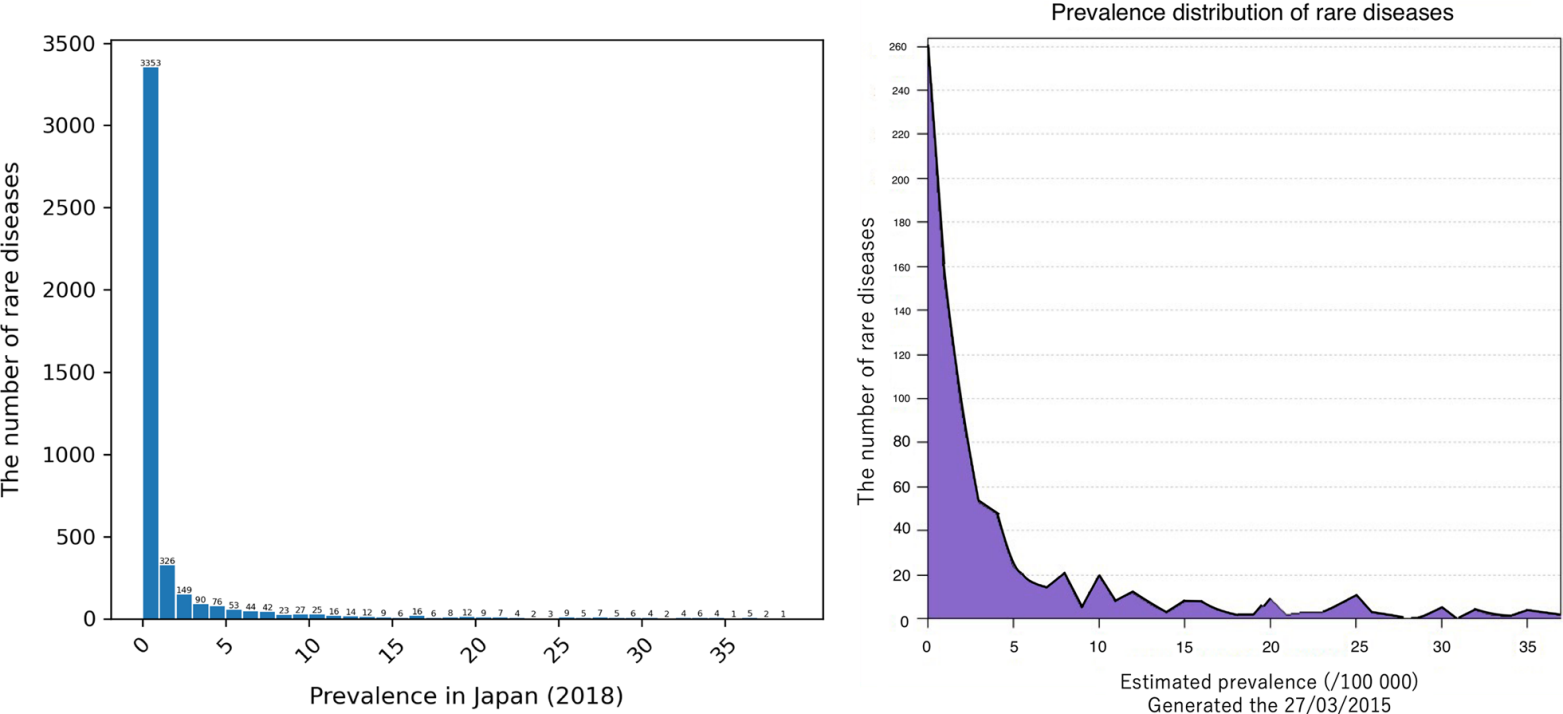
Comparison of prevalence distribution of rare diseases in NDB and Orphanet data. The preavalences are calcurated per 100,000 persons as in the Orphanet report. As more recent data tends to be more accurate, the figure of NDB is generated based on the data in the most recent year, 2018

We chose rare diseases from the ICD10-based Standard Disease-Code Master through a semiautomatic process to follow NDB regulations. In Fig. 9, we roughly compare the prevalence distribution in NDB with that in Orphanet to validate the disease selection. They show the same trend, indicating that most rare diseases affect only several persons per 100,000. Moreover, Fig. 3 demonstrates that more than 60% of the diseases in NDB have equivalent terms in other rare disease databases. Therefore, it is our informed opinion that the selected diseases can mostly be recognized as rare.

### Prevalence

To reveal the difference between the prevalence of each disease in NDB and that in Orphanet, we compared them by discretizing the actual prevalence value in NDB. Figure 4 shows that the difference of about 80% of the diseases is only within one category. It is also revealed that most diseases belong to less than 1–9/100,000. If we pay attention to the Japanese data, a difference in one category means practically less than about one error for 1000 Japanese patients. Accordingly, the analysis implies only a slight discrepancy between NDB data and Orphanet data.

If we focus on the relatively small difference, given that the categories of Orphanet tend to be higher, there seem to be two possible explanations. First, as Orphanet mainly contains prevalence in European countries and most diseases with a categorical difference are genetic, it could reflect an ethnic group difference. The other is that rare diseases in many Japanese patients remain undetected. The latter reason can be supported by the fact that there are much fewer genetic tests available in Japan than there are in the West [30]. On the other hand, if we look closely at the relatively significant difference, 11 diseases indicate a higher category in Orphanet by more than four categories. This tendency might be due to excessive regional accumulation, such as in the cases of schistosomiasis and Cooley anemia.

As for the comparison between Japanese prevalence in Orphanet and NDB, Fig. 5 shows the same results as aforementioned. As most diseases are within one category’s difference, the Orphanet data are consistent with the NDB data.

### Natural history

According to Figs. 7 and 8, high-density areas seem to accord with the Orphanet categories, predefined on the basis of age distribution patterns. Therefore, the natural history of most diseases in NDB is consistent with that in Orphanet in general.

However, some diseases indicate different natural histories in Japan. This could happen because of the unique therapeutic environment available in each country; clinical variability caused by ethnic factors, such as genetic variation and diet; and artifacts accompanied by time-series clustering. To focus on the apparent distinction of natural history, we calculated the number of diseases with more than two categorical differences, excluding those with the “All ages” onset category, “normal life expectancy” death category, and “any age” death category (Tables 1, 2).

**Table 1:** The differences in the average age of onset in NDB and Orphanet

NDB	Orphanet	Number of diseases
*Case 1. Average age of onset is later in NDB than that in Orphanet*		
Acquired	Antenatal, neonatal, infancy	21
Elderly	Antenatal, neonatal, infancy, childhood	80
*Case 2. Average age of onset is earlier in NDB than that in Orphanet*		
Congenital	Adolescent, adult, elderly	4

**Table 2:** Differences in the average age of death in NDB and Orphanet

NDB	Orphanet	Number of diseases
*Case 3. Average age of death is later in NDB than that in Orphanet*		
Childhood	Stillbirth, infantile	1
Acquired	Infantile, adolescent, late childhood	2
Elderly	Infantile, stillbirth, early childhood, adolescent	8
*Case 4. Average age of death is earlier in NDB than that in Orphanet*		
Congenital	Adult, elderly	4
Childhood	Elderly	1

Tables 1 and 2 show the disagreement between NDB-based age distribution patterns and Orphanet categories of the average age of onset and the average age of death, respectively. As some diseases have multiple categories in Orphanet, we defined coincidence between two databases as the condition for more than one Orphanet category to be included in the range of the NDB category.

In Case 1, Japanese patients tend to present symptoms later than those in the Orphanet data do. About two-thirds of the diseases are genetic, and only 30% are covered by the Nanbyo system for children. Moreover, as mentioned above, genetic tests of numerous diseases are not available in Japan. This seems to relate to the delayed detection of Japanese patients. On the other hand, Case 2 indicates that the average onset age of the four diseases is earlier in NDB than it is in Orphanet. All diseases, except encephalitis lethargica, are categorized as cancer, and the number of patients demonstrates a bimodal distribution. As our time-series clustering could not catch bimodality, we observed this difference.

Case 3 implies that Japanese patients live longer than those in the Orphanet data do. This seems due to the bimodal distribution of diseases that can be secondary, such as biliary atresia. Conversely, in Case 4, five diseases in all, Bloom syndrome, achondroplasia, 4p deletion syndrome, Dent disease, and hereditary pancreatitis, show earlier death. Although it is difficult to determine the reason, this may be due to the variation of their severity or phenotypes and their therapeutic environment.

### Japanese policy on rare diseases

Although Japan has provided medical expense subsidies for Nanbyo patients, fairness between rare diseases has always been controversial. That is to say, the increasing number of rare diseases casts doubt on the legitimacy of a dividing line between Nanbyo and non-Nanbyo rare diseases although several diseases are added to Nanbyo almost every year. In fact, there are some procedures and obstacles to having a disease covered by the Nanbyo system. First, an official research group focusing on a specific disease is required to be established. Its role is to set diagnostic criteria or a guideline and reveal its prevalence, mainly through mail questionnaire surveys of medical institutions all over the country. Then, the advisory board of each Nanbyo system discusses whether it should be added to the system on the basis of its report.

Consequently, only a few diseases pass after receiving public comments every year. A serious problem, in particular, among various concerns, is that as research groups can only focus on limited rare diseases, it depends on chance or the degree of disease recognition at best, whether a rare disease is designated as Nanbyo and its patients can receive medical support. Accordingly, ultra-rare diseases in particular have hardly any opportunities to be examined.

As aforementioned, one of the requirements for designation is that the number of patients with a specific disease be less than 120,000, which indicates 0.1% of the Japanese population. In light of this regulation, Fig. 2 suggests that approximately 4000 diseases are supposed to be covered, which means newly adding 3000 diseases. Moreover, even if the Japanese government comprehensively adopts the Orphanet criteria to meet global standards, Fig. 3 implies that about 800 diseases are left outside the coverage of the Nanbyo systems. Strictly speaking, as Nanbyo is not always included in Orphanet, 1085 non-Nanbyo diseases recognized as rare afflict patients as per Orphanet. Therefore, despite the unique circumstances surrounding Japan’s healthcare system, it is evident that Nanbyo does not cover enough rare diseases.

To ensure fairness, Japanese policies need to promote active comprehensive research to detect rare-disease patients, referring to the medical databases in the world, and become more flexible in providing medical expense subsidies. In addition, even if a specific disease is inside the system, patients afflicted by it need to be strictly examined for eligibility for medical support based on objective criteria, such as phenotypes, disease severity, medical costs, and quality of life, although this does not easily seem acceptable to patients who have already been designated as eligible. Accordingly, these enable a significant turnover of patients eligible for the support and sustainability of Nanbyo systems.

### Limitations

As NDB was not originally constructed for analysis, it presents several limitations in its use. First, as diseases in the NDB data are standardized to meet the procedural needs of clinical practice in Japan, our NDB data could inevitably be incomplete according to the definition of rare diseases in the world. The shortage of rare diseases is mainly because they are too rare in Japan to be added to the code master. In addition, as we semi-automatically chose them from 23,939 diseases, we could have overlooked or excessively detected diseases. Therefore, it is still challenging to thoroughly elucidate the circumstances of rare-disease patients in Japan.

Second, the system of health insurance claims and procedures complicated the interpretation of the NDB data. Some patients are exempt from any medical expense, such as welfare recipients, atomic bomb survivors, and people under the severe condition of insanity. It is sometimes challenging for persons with rare, severe diseases to work. In such cases, the NDB data do not contain their information. Moreover, it is said that 80% of diseases labeled “other diseases” in the code master are wrongly allocated although there are correct categories for them [31]. Consequently, the number of patients seems underestimated. On the other hand, its overestimation can occur because doctors often make a temporary diagnosis stored in the NDB to prescribe common treatments such as analgesics under health insurance. However, this is primarily true of common diseases; hence, this artifact has little effect on the interpretation of our data. Another confusing factor is that both disease and subtype are coded in the master. For example, as Niemann–Pick disease and Niemann–Pick disease type A are on the list, it is nearly impossible to know whether patients are counted conflatingly or exclusively, which depends on doctors or clinical coders.

The last limitation is that in calculating prevalence, we might have underestimated it because we adopted the entire population of Japan as a denominator instead of the total population in the NDB data, assuming that almost all the people receive medical treatment under health insurance more than once a year.

## Conclusion

Our research revealed the epidemiology of Japanese patients with rare diseases using a health insurance claims database. In particular, this study is the first to shed light on the presence of rare diseases outside of the Nanbyo systems. The results imply that the present Nanbyo systems do not cover enough rare diseases. Therefore, they may require drastic reform to reduce unfairness between rare diseases even if it is a difficult decision for some people.

We also elucidated the natural history of rare diseases among the Japanese population. It primarily indicates a similar tendency to that reported in Orphanet. However, we found that some diseases are detected later in Japan. As a systemic flaw of clinical genetic tests may cause this result, we expect the new system that has been discussed to be established in the not-too-distant future.

As people exempt from medical expenses have been included in NDB since December 2021 and multiple databases focusing on rare diseases was disclosed by the ministry in 2020, futher research is needed to reveal more detailed actual conditions of patients in Japan, such as therapeutic interventions, quality of life, and social participation.

Lastly, we hope that our data and analysis provides patients with improved access to much needed treatments in Japan and ultimately give courage to those patients who feel lonely and helpless.

## Supplementary Information

**Additional file 1**. The tabular datasets derived from the National Database of Health Insurance Claims and Specific Health Checkups of Japan.

## Data Availability

The tabular datasets supporting the conclusions of this article are available in the Additional file 1, the website of Japanese Rare Diseases Research Consortium (JRDiRC), and NanbyoData. JRDiRC is a semi-closed community of people concerned with rare diseases in Japan to exchange information (https://member.jrdirc.org/). As NanbyoData is a website aimed at providing accumulated multilingual information on rare diseases in Japan, it provides the data with the direct links between Nanbyo and rare diseases in databases on various medical fields (https://nanbyodata.jp).

## Abbreviations

NDB: National Database of Health Insurance Claims and Specific Health Checkups of Japan
ICD: International Statistical Classification of Diseases and Related Health Problems
NANDO: Nanbyo Disease Ontology
